# Recent Trends in Pharmaceutical Radiochemistry for Molecular PET Imaging

**DOI:** 10.1155/2014/890540

**Published:** 2014-07-24

**Authors:** Olaf Prante, Roland Haubner, Patrick Riss, Bernd Neumaier

**Affiliations:** ^1^Molecular Imaging and Radiochemistry, Department of Nuclear Medicine, Friedrich-Alexander University Erlangen-Nürnberg (FAU), Erlangen, Germany; ^2^Department of Nuclear Medicine, Medical University Innsbruck, Innsbruck, Austria; ^3^Department of Chemistry, University Oslo, Oslo, Norway; ^4^Institute of Radiochemistry and Experimental Molecular Imaging, University Clinic Cologne and Max Planck Institute of Metabolic Research, Cologne, Germany

In the field of radiopharmaceutical research, the development of new radiochemistry methods has been one of the major driving forces for positron emission tomography (PET) imaging during the past decade. The use and availability of the positron emitters C-11, F-18, Ga-68, Cu-64, or Zr-89, to name a few, have enormously increased and, especially in terms of chemoselectivity and radiolabeling efficacy, significant progress has been made. In the field of F-18 chemistry, various click chemistry-based labeling methods, the use of the silicon-fluoride acceptor reagents, and Al-F-NOTA complexes offer an even more simplified strategy to introduce F-18 into biomolecules. These techniques facilitate the syntheses of radiotracers for PET imaging studies and thus accelerate their pronounced use in preclinical studies and even clinical trials. A similar situation is seen in the field of metallic positron emitters, where additional strategies have been developed to extend and to improve radiometal chemistry, for example, by introducing Zr-89 for the labeling of antibodies and long-term imaging studies.

The field of radiopharmaceutical sciences has been mainly influenced by its founders and their pioneering work. One of the scientific pioneers of modern radiochemistry for imaging by PET, Professor Heinz H. Coenen, is celebrating his 65th birthday the same time this special issue was published. He is highly recognized in the field of radiochemistry and molecular imaging and one of the authors of the most cited paper in the field of nuclear medicine and molecular imaging. He has been director of the German Research Center in Jülich for more than 15 years and has been international president of the largest radiopharmaceutical society (Society of Radiopharmaceutical Sciences, SRS).

This special issue describes many important and recent research advancements in PET chemistry that have been influenced by the pioneering work of Professor Heinz H. Coenen. Additionally, this special issue is thought to create awareness of multiple imaging applications of newly developed radiotracers and thereby encourages young researchers to expand their projects and developments by applying these modern techniques. However, it is clearly impossible in an issue of this size to cover all recent developments in PET chemistry.

We do not pretend to be infallible in collecting review papers with such a wide variety of topics. Some of the articles in this special issue were written by former Ph.D. degree students of Professor Coenen and we are sure that especially these research topics were significantly inspired and motivated by their early scientific work together with Professor Heinz H. Coenen.

We were pleased that Professor Bernd Neumaier, whose scientific mentor has been Professor Heinz H. Coenen, agreed to perform some comments on the different contributions in this special issue.

*Olaf Prante* *Roland Haubner* *Patrick Riss*

**Foreword by Bernd Neumaier (Institute of Radiochemistry and Experimental Molecular Imaging and Max Planck Institute for Neurological Research, Cologne, Germany)**

Broad application of noninvasive imaging techniques, especially positron emission tomography (PET) and related hybrid methods (PET/CT and PET/MR), in clinical practice has significantly contributed to a considerabe increase of accuracy in clinical diagnostics. PET offers quantitative 3D-visualization of physiological and pathological processes* in vivo* using probes labeled with positron-emitting nuclides. Moreover, PET represents a powerful tool for drug development which allows precise assessment and validation of their pharmacological properties at a molecular level. Furthermore, novel PET-tracers enable monitoring the success of anticancer treatment. The consistent growth of PET is accompanied by a large unmet need for the development of novel PET-probes including labeling techniques for the visualization of suitable targets of various diseases.

The starting point of modern PET imaging was the introduction of 2-[^18^F]fluoro-2-deoxy-D-glucose ([^18^F]FDG) in clinical practice (1976). However, highly sophisticated preparation procedures prevented its widespread application. This situation changed entirely after the introduction of an efficient stereospecific synthesis of n.c.a. [^18^F]FDG using aminopolyether supported nucleophilic ^18^F-substitution proposed by Kurt Hamacher, Heinz H. Coenen, and Gerhard Stöcklin in 1986. The novel radiosynthesis enabled obtaining [^18^F]FDG in amounts allowing its broad clinical application. Moreover, this radiofluorination method has an enormous impact on ^18^F-chemistry until today. That is one of the numerous trend-setting works of Professor Heinz H. Coenen, whose concepts influenced radiochemistry substantially. Although his scientific work covers different aspects of nuclear chemistry his achievements in modern ^18^F-labeling chemistry are of exceptional importance. His work on the preparation of ^18^F-PET-tracers from iodonium salts as well as the production of ^18^F-labeled amino and fatty acids and their application for tumor imaging are definitely further highlights of his work. Accordingly, the present issue is dedicated to Professor Heinz H. Coenen on the occasion of his 65th birthday. Not surprisingly, in this issue excerpts of his pioneering works can be found.

The majority of papers in this issue deal with ^18^F radiolabeling chemistry reflecting the outstanding potential of ^18^F in PET imaging. The exceptional position of this radioisotope is based on its favorable nuclear properties (half-life and *β* ^+^-decay) and easy accessibility in >50 GBq quantities.

In recent years copper-catalyzed azide-alkyne click reactions have become a convenient method to introduce ^18^F under mild reaction conditions. Further developments make use of more reactive 1,3-dipoles beyond azide and/or exploit strain-promoted metal-free click chemistry to prepare radiofluorinated compounds. This topic is reviewed by K. Kettenbach et al.

Recently, the silicon-fluoride-acceptor isotopic exchange (SIFA-IE) was established for ^18^F-labeling. This novel approach gives rise to objective advantages such as no need for separation of radiolabeled product from precursor and very mild reaction conditions. The works in this field are covered by the contribution of V. Bernard-Gauthier et al.

Further, this issue provides a deeper insight into the radiosynthesis of small ^18^F-molecules as intermediates for modular build-up syntheses. A plethora of labeling methods for the synthesis of ^18^F-labeled building blocks for the construction of radiofluorinated complex molecules is reviewed by J. Ermert.

The CF_3_ moiety is present in a large number of pharmaceuticals and drug candidates. The introduction of the trifluoromethyl group is often applied to improve pharmacological properties of lead structures. Consequently, several methods for introduction of 2-[^18^F]fluoro-2,2-difluoroethyl group in target molecules have been proposed. They are presented in detail by V. T. Lien and P. J. Riss.

Chemoselective ^18^F-fluoroglycosylation, for example, via azide-alkyne click reactions or via oxime formation allows preparing structurally defined ^18^F-labeled glycoconjugates which often display improved* in vivo* kinetics and increased metabolic stability compared to parent compounds. S. Maschauer and O. Prante give a brief overview of the developments in this emerging field.

^18^F-Chemistry is topped off with a review on 6-l-[^18^F]FDOPA, the most popular PET-neurotracer with an exceptionally broad spectrum of applications. The paper of M. Pretze et al. summarizes the developments in the field of [^18^F]F-DOPA syntheses using electrophilic synthesis pathways as well as recent developments of nucleophilic syntheses of 6-l-[^18^F]FDOPA and compares the different synthesis strategies regarding the accessibility and applicability of the products for human* in vivo* PET tumor imaging.

Radiolabeled RGD peptides are of great importance for tumor detection since overexpression of definite integrins is frequently associated with tumor-induced angiogenesis and tumor metastasis. The contribution of R. Haubner et al. deals with different labeling techniques for the production of radiolabeled RGD-peptides. Beside different ^18^F-labeling methods, an overview of other opportunities to efficiently label RDG peptides is provided. Furthermore, novel sequences targeting other integrin subgroups such as *α*_5_ *β*_1_ are described.

Owing to very slow blood clearance and metabolism of antibodies conventional PET emitters are unsuitable for PET measurements. This problem can be overcome, for example, by using the long-lived positron emitter ^89^Zr. Strategies for ^89^Zr-labeling of antibodies and use of ^89^Zr-labeled antibodies for PET-imaging are outlined by F. C. J. van de Watering et al.

Hybrid imaging technologies which combine different imaging modalities can provide additional clinical advantages. Some of them such as PET/CT and PET/MR are already widely applied in clinics. Despite its great potential, the combination of PET with optical imaging (OI) still remains in the phase of preclinical development. The paper authored by U. Seibold et al. is devoted to the preparation of and preclinical feasibility studies with bimodal agents for PET/OI imaging.

The current issue has not been designed to be comprehensive but, instead, to demonstrate the versatility, dynamics, and challenges of modern PET-chemistry. The efforts in this fast growing field aim at a steady improvement of existing and development of novel radiolabeling procedures in order to actively implement the “from bench to bedside” approach and, ultimately, to improve patient care.

*Bernd Neumaier*

